# Population attributable risk estimates of risk factors for contrast-induced acute kidney injury following coronary angiography: a cohort study

**DOI:** 10.1186/s12872-020-01570-6

**Published:** 2020-06-12

**Authors:** Li Lei, Yan Xue, Zhaodong Guo, Bowen Liu, Yibo He, Feier Song, Jin Liu, Guoli Sun, Liling Chen, Kaihong Chen, Zhiqi Su, Li Pan, Zhidong Huang, Yulu Huang, Xiuqiong Huang, Shiqun Chen, Jiyan Chen, Yong Liu

**Affiliations:** 1 The Second School of Clinical Medicine, Southern Medical University, Guangzhou, 510515 Guangdong China; 2 Department of Cardiology, Provincial Key Laboratory of Coronary Heart Disease, Guangdong Cardiovascular Institute, Guangdong Provincial People’s Hospital, Affiliated Guangdong Provincial People’s Hospital of South China University of Technology, Guangdong Academy of Medical Sciences, Guangzhou, 510080 Guangdong China; 3 Department of Cardiology, the People’s Hospital of Guangxi Zhuang Autonomous Region, Nanning, Guangxi China; 4 Guangdong Provincial People’s Hospital, School of Medicine, South China University of Technology, Guangzhou, Guangdong China; 5 Department of Emergency and Critical Care Medicine, Guangdong Provincial People’s Hospital and Guangdong Academy of Medical Sciences, Guangzhou, Guangdong China; 6 Department of Cardiology, Longyan First Affiliated Hospital of Fujian Medical University, Longyan, 364000 Fujian China; 7 School of Pharmacy, Guangdong Pharmaceutical University, Guangzhou, Guangdong China

**Keywords:** Catheterization, Acute renal disease, Risk factors, Population attributable risk

## Abstract

**Background:**

Contrast-induced acute kidney injury (CI-AKI) is a common complication with poor outcomes following coronary angiography (CAG) or percutaneous coronary intervention (PCI). However, no study has explored the population attributable risks (PARs) of the CI-AKI risk factors. Therefore, we aimed to identify the independent risk factors of CI-AKI and estimate their PARs.

**Methods:**

We analyzed 3450 consecutive patients undergoing CAG/PCI from a prospective cohort in Guangdong Provincial People’s Hospital. CI-AKI was defined as a serum creatinine elevation ≥50% or 0.3 mg/dL from baseline within the first 48 to 72 h after the procedure. Independent risk factors for CI-AKI were evaluated through stepwise approach and multivariable logistic regression analysis, and those that are potentially modifiable were of interest. PARs of independent risk factors were calculated with their odds ratios and prevalence among our cohort.

**Results:**

The overall incidence of CI-AKI was 7.19% (*n* = 248), which was associated with increased long-term mortality. Independent risk factors for CI-AKI included heart failure (HF) symptoms, hypoalbuminemia, high contrast volume, hypotension, hypertension, chronic kidney disease stages, acute myocardial infarction and age > 75 years. Among the four risk factors of interest, the PAR of HF symptoms was the highest (38.06%), followed by hypoalbuminemia (17.69%), high contrast volume (12.91%) and hypotension (4.21%).

**Conclusions:**

These modifiable risk factors (e.g., HF symptoms, hypoalbuminemia) could be important and cost-effective targets for prevention and treatment strategies to reduce the risk of CI-AKI. Intervention studies targeting these risk factors are needed.

## Background

Contrast-induced acute kidney injury (CI-AKI) is a common complication of coronary diagnostic and interventional procedures that is significantly associated with the composite endpoint of major adverse renal and cardiovascular events (MARCE) [1–3]. The 2018 European Society of Cardiology (ESC)/European Association for Cardio-Thoracic Surgery (EACTS) Guidelines on myocardial revascularization recommend assessing all patients for the risk of CI-AKI [4]. Screening and identifying patients at risk of CI-AKI would easily and accurately allow prophylactic intervention in those at high risk. Research on the prevention of CI-AKI has focused on the use of intravenous fluids, renal replacement therapies (RRTs), and pharmaceutical agents. One of the challenges in clinical practice is that the population benefits of preventive RRT and drug treatments have not been proved [1, 2]. Clinicians do not have enough information to improve evidence-based screening and prevention efforts.

The population attributable risk (PAR) represents the proportion of disease cases in a population that would not have occurred in the absence of a risk factor [5]. Many observational studies regarding CI-AKI risk factors have been reported, but to our knowledge, no studies quantifying the contributions of risk factors of CI-AKI have been identified [6]. There is a continued need to identify which risk factors have the greatest impact on CI-AKI, especially those that might be potentially modifiable by interventions.

Therefore, we aimed to evaluate the association between several risk factors commonly documented in clinical practice and CI-AKI and to estimate their PARs among a large prospective cohort.

## Methods

### Patient selection

The 3450 consecutive patients undergoing coronary angiography (CAG) or percutaneous coronary intervention (PCI) between January 2010 and October 2012 in Guangdong Provincial People’s Hospital were enrolled. The exclusion criteria included contrast exposure within the previous 7 days or 3 days after the procedure, pregnancy, lactation, cardiovascular surgery, no use of low-osmolarity contrast agents, undergoing hemodialysis, missing preoperative or postoperative creatinine, malignancy, and no use of isotonic saline for hydration [7]. The study was approved by the Ethics Committee of Guangdong Provincial People’s Hospital. All the patients included in this study signed written informed consent.

### Endpoint and definitions

The primary endpoint of this study was CI-AKI, defined as a serum creatinine (Scr) elevation ≥50% or 0.3 mg/dL from baseline within the first 48 to 72 h after the procedure. The secondary endpoint was all-cause death. All eligible patients included were followed up through office visits or telephone interviews at 1 month, 6 months and every year after enrollment until April 2019. High contrast volume was defined as contrast volume > 155 mL during the procedure. The cutoff value of “155 mL” to determine high contrast volume was derived from the receiver operating characteristic curve that had the maximal sum of sensitivity and specificity [8]. Heart failure (HF) symptoms were defined as New York Heart Association (NYHA) class > I/Killip class > I on presentation. Chronic kidney disease (CKD) was defined as estimated glomerular filtration rate (eGFR) < 60 mL/min/1.73 mm^2^. Patients with CKD were also divided into 3 stages (mild: eGFR: 45–60 mL/min/1.73 mm^2^; moderate: eGFR: 30–45 mL/min/1.73 mm^2^; severe: eGFR: < 30 mL/min/1.73 mm^2^) [9]. Hypoalbuminemia was defined as serum albumin (ALB) < 35 g/L [10]. Anemia was defined as hematocrit < 36% for women and < 39% for men, and hypotension was defined as systolic blood pressure < 80 mmHg for at least 1 h requiring inotropic support with medications or intra-aortic balloon pump (IABP) within 24 h peri-procedurally [11]..

### Study protocol

The procedure was performed according to published guidelines [12]. The contrast volume was determined by the operators. A noninvasive treatment strategy was performed according to published guidelines and clinical routines. Scr was measured for all patients at admission and at 1, 2, and 3 days after the procedure.

### Statistical analysis

Risk factors of interest (HF symptoms, hypoalbuminemia, high contrast volume, hypotension) that are potentially modifiable and independently associated with CI-AKI were selected based on the result of multivariable logistic regression, previous studies and clinical importance [1].

For continuous variables, data are expressed as mean ± standard deviation and compared between 2 groups through the independent samples t-test if they were normally distributed; otherwise, data are expressed as median ± interquartile range and compared between groups using the Wilcoxon rank-sum test. For categorical data (expressed as percentages), Pearson’s chi-squared or Fisher’s exact test was conducted. Long-term mortality in patients with or without CI-AKI was assessed with Kaplan-Meier survival curves, and equality tests of survival distributions were compared using the log-rank test. Moreover, we did landmark analyses to assess all-cause mortality at 90-days and after 90-days.

Univariable logistic regression was conducted for risk factors that were imbalanced between groups and with missing value < 15%. For variables that had interaction or collinearity between each other, the modifiable or categorical one was preferred for better clinical implication. Factors with significant impotence in univariable logistic regression were then enrolled in a backward stepwise approach which successively removing non-significant covariates (*P* > 0.05) until all the remaining predictors are statistically significant. Multivariable logistic regression model including all the remaining risk factors were then fitted to calculate the odds ratios (ORs) for their impact on CI-AKI. PAR was calculated for independent risk factors using the equation PAR = P (OR-1)/[1 + P (OR-1)], where P is the prevalence of each risk factor in our database. The standard error of PAR was calculated using the delta method [13]. All data analyses were conducted with R software (version 3.6.2; R Foundation for Statistical Computing, Vienna, Austria).

## Results

### Baseline characteristics

All 3450 eligible patients were included in the final analysis, among whom the incidence rate of CI-AKI was 7.19% (*n* = 248). Table 1 details the patient characteristics. Patients with CI-AKI following CAG were older and emaciated. They had higher proportion of impaired heart and renal function, hypertension, hypoalbuminemia, anemia, acute myocardial infarction (AMI) and coronary artery disease (CAD). Higher heart rate, C-reactive protein and serum urea nitrogen were also identified in those complicated with CI-AKI. During their hospitalization, they were more likely to be prescribed diuretics and antibiotics. Higher dose of contrast media during the procedure was also found in those with CI-AKI.

**Table 1:** Baseline characteristics of patients with or without contrast-induced acute kidney injury

Variables	Total (*n* = 3450)	No. (%) of patients with available data	CI-AKI group (*n* = 248)	Non-CI-AKI group (*n* = 3202)	*P* value
Age, y	62.94 ± 11.13	3450 (100)	69.11 ± 11.13	62.45 ± 10.99	< 0.001
Age > 75, n (%)	571 (16.55)	3450 (100)	89 (35.89)	482 (15.05)	< 0.001
Female sex, n (%)	802 (23.25)	3450 (100)	67 (27.02)	735 (22.95)	0.145
Weight, kg	64.89 ± 10.75	3419 (99.10)	62.38 ± 10.71	65.07 ± 10.73	< 0.001
SBP, mmHg	128.85 ± 20.48	3439 (99.68)	127.78 ± 25.55	128.93 ± 20.04	0.492
DBP, mmHg	75.95 ± 11.91	3438 (99.65)	74.74 ± 12.74	76.04 ± 11.84	0.123
HR, bpm	75.07 ± 13.42	3437 (99.62)	79.81 ± 17.07	74.71 ± 13.03	< 0.001
Medical history					
CAD, n (%)	3077 (89.76)	3428 (99.36)	234 (95.12)	2843 (89.34)	0.004
Chronic heart failure, n (%)	1961 (56.99)	3441 (99.74)	182 (73.39)	1779 (55.72)	< 0.001
CKD, n (%)	643 (18.64)	3450 (100)	114 (45.97)	529 (16.52)	< 0.001
CKD stages					< 0.001
Mild CKD, n (%)	397 (11.51)		50 (20.16)	347 (10.84)	
Moderate CKD, n (%)	177 (5.13)		35 (14.11)	142 (4.43)	
Severe CKD, n (%)	69 (2.00)		29 (11.69)	40 (1.25)	
Hypotension, n (%)	89 (2.58)	3445 (99.86)	28 (11.43)	61 (1.91)	< 0.001
LVEF, %	57.78 ± 12.26	3008 (87.19)	51.17 ± 12.90	58.32 ± 12.05	< 0.001
LVEF < 40%, n (%)	294 (9.77)	3008 (87.19)	43 (18.94)	251 (9.03)	< 0.001
HF symptoms, n (%)	1876 (54.85)	3420 (99.13)	170 (68.55)	1706 (53.78)	< 0.001
Hypertension, n (%)	1962 (56.89)	3449 (99.97)	172 (69.35)	1790 (55.92)	< 0.001
Hyperlipidemia, n (%)	507 (14.70)	3450 (100)	27 (10.89)	480 (14.99)	0.079
Smoking, n (%)	1371 (39.74)	3450 (100)	89 (35.89)	1282 (40.04)	0.198
Hypoalbuminemia, n (%)	1334 (44.78)	2979 (86.35)	126 (69.61)	1208 (43.17)	< 0.001
Anemia, n (%)	1086 (31.81)	3414 (98.96)	116 (47.15)	970 (30.62)	< 0.001
AMI, n (%)	1300 (37.85)	3435 (99.57)	163 (65.99)	1137 (35.66)	< 0.001
Diabetes, n (%)	817 (23.69)	3448 (99.94)	71 (28.63)	746 (23.31)	0.058
Laboratory measurements					
LDL-C, mmol/L	2.74 ± 0.97	2874 (83.30)	2.97 ± 1.01	2.72 ± 0.97	0.001
HDL-C, mmol/L	1.00 ± 1.30	2872 (83.25)	0.99 ± 0.27	1.00 ± 1.34	0.682
NTpro-BNP, pg/mL	1374.48 ± 3404.48	2326 (67.42)	5442.54 ± 8475.37	1092.05 ± 2492.00	< 0.001
Hs-CRP, mg/L	17.49 ± 33.91	2644 (76.64)	42.16 ± 50.94	15.57 ± 31.42	< 0.001
Lpa, mg/dL	30.68 ± 34.43	3037 (88.03)	31.28 ± 33.54	30.63 ± 34.50	0.785
SCR, μmol/L	92.35 ± 42.37	3450 (100)	117.14 ± 58.53	90.42 ± 40.23	< 0.001
eGFR, mL/min/1.73 mm^2^	80.95 ± 25.55	3450 (100)	67.81 ± 36.71	81.97 ± 24.18	< 0.001
Serum urea nitrogen, mg/dL	5.29 ± 2.55	3414 (98.96)	6.85 ± 3.73	5.17 ± 2.40	< 0.001
Hemoglobin, g/L	132.98 ± 16.35	3072 (89.04)	124.88 ± 20.52	133.52 ± 15.89	< 0.001
HbA1c, %	6.54 ± 1.32	2648 (76.75)	6.78 ± 1.50	6.52 ± 1.31	0.024
Serum albumin, g/L	35.23 ± 7.04	2979 (86.35)	32.34 ± 4.58	35.42 ± 7.13	< 0.001
Medications					
ACEI/ARB, n (%)	3019 (87.53)	3449 (99.97)	201 (81.38)	2818 (88.01)	0.002
Beta blocker, n (%)	2919 (84.63)	3449 (99.97)	176 (70.97)	2743 (85.69)	< 0.001
Statin, n (%)	3314 (96.09)	3449 (99.97)	233 (94.33)	3081 (96.22)	0.140
Diuretics, n (%)	654 (18.96)	3449 (99.97)	118 (47.58)	536 (16.74)	< 0.001
Antibiotic, n (%)	527 (17.11)	3080 (89.28)	73 (39.25)	454 (15.69)	< 0.001
CCB, n (%)	594 (17.26)	3442 (99.77)	37 (15.10)	557 (17.42)	0.354
PPI, n (%)	1456 (42.30)	3442 (99.77)	154 (62.60)	1302 (40.74)	< 0.001
Metformin, n (%)	81 (2.63)	3079 (89.25)	2 (1.08)	79 (2.73)	0.236
Procedure					
PCI, n (%)	2087 (67.65)	3085 (89.42)	143 (76.06)	1944 (67.10)	0.011
Contrast volume, mL	126.53 ± 64.54	3447 (99.91)	135.44 ± 64.34	125.84 ± 66.50	< 0.001
Contrast volume > 155, n (%)	852 (24.72)	3447 (99.91)	80 (32.26)	772 (24.13)	0.004
Peri-procedure IABP, n (%)	133 (3.86)	3450 (100)	57 (22.98)	76 (2.37)	< 0.001

Abbreviations: *CI-AKI* contrast-induced acute kidney injury, *SBP* systolic blood pressure, *DBP* diastolic blood pressure, *HR* heart rate, *LVEF* left ventricular ejection fraction, *HF* heart failure, *CKD* chronic kidney disease, *AMI* acute myocardial infarction, *LDL-C* low-density lipoprotein-C, *HDL-C* high-density lipoprotein-C, *HS-CRP* high-sensitivity C-reactive protein, *SCR* serum creatinine, *Lpa* lipoprotein a, *eGFR* estimated glomerular filtration rate, *ACEI* angiotensin-converting enzyme inhibitor, *ARB* angiotensin-receptor blockers, *PPI* proton pump inhibitors, *CCB* calcium channel blocker, *PCI* percutaneous coronary intervention, *CAD* coronary artery disease, *IABP* intra-aortic balloon pump

### The association between CI-AKI and prognosis

After the procedure, 26 (10.48%) patients who were complicated with CI-AKI underwent hemodialysis, while 8 (0.25%) patients without CI-AKI underwent hemodialysis (*P* <  0.001).

**Fig. 1:**
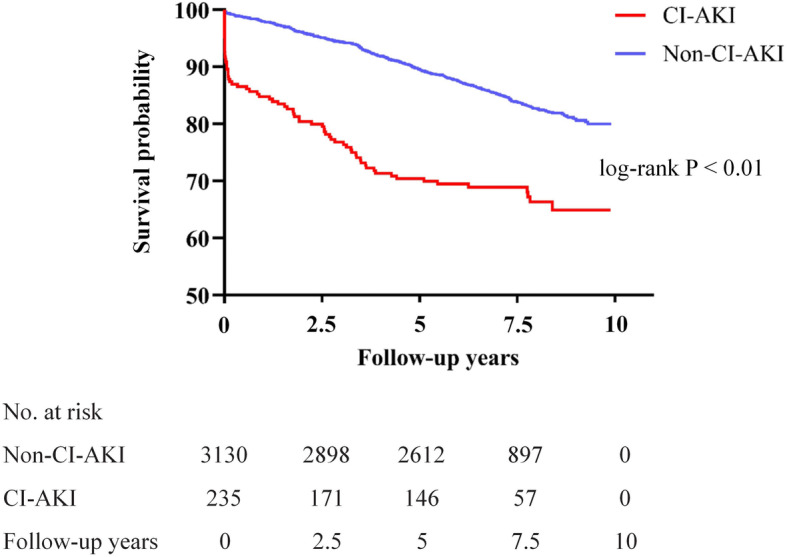
Association between contrast-induced acute kidney injury and long-term survival in patients `undergoing coronary angiography. Abbreviations: CI-AKI: contrast-induced acute kidney injury

During the median follow-up of 7.41 (6.21; 8.27) years, mortality was 17.0% (*n* = 586) in total, 31.9% (*n* = 79) in patients with CI-AKI, and 15.8% (*n* = 507) in patients without CI-AKI (*P* <  0.001). Kaplan-Meier survival curves revealed that patients with CI-AKI following CAG had a higher mortality rate than those without CI-AKI (log-rank *P* <  0.01; Fig. 1). The significant association between CI-AKI and all cause death at 90-days follow-up was maintained after 90-days. (Figure S1).

### Risk factors for CI-AKI

Multivariable logistic regression revealed that hypoalbuminemia (OR: 1.48, 95% CI: 1.03–2.13), HF symptoms (OR: 2.12, 95% CI: 1.46–3.06), high contrast volume (OR: 1.60, 95% CI: 1.14–2.24), hypotension (OR: 2.70, 95% CI: 1.06–6.86), hypertension (OR: 1.45, 95% CI: 1.01–2.08), mild CKD (OR: 2.23, 95% CI: 1.46–3.40), moderate CKD (OR: 3.39, 95% CI: 2.05–5.62), severe CKD (OR: 6.95, 95% CI: 3.48–13.90), AMI (OR: 3.24, 95% CI: 2.29–4.58) and age > 75 years (OR: 2.02, 95% CI: 1.41–2.88) were independently associated with CI-AKI (Table 2).

**Table 2:** Univariable and multivariable logistic regression for risk factors of contrast-induced acute kidney injury

	Univariable		Multivariable	
Variables	OR (95% CI)	*P* value	OR (95% CI)	*P* value
Hypoalbuminemia	3.02 (2.18–4.18)	< 0.001	1.48 (1.03–2.13)	0.033
HF symptoms	1.87 (1.42–2.47)	< 0.001	2.12 (1.46–3.06)	< 0.001
High contrast volume	1.50 (1.13–1.98)	0.005	1.60 (1.14–2.24)	0.006
Hypotension	6.64 (4.16–10.60)	< 0.001	2.70 (1.06–6.86)	0.037
Age > 75 years	3.16 (2.40–4.17)	< 0.001	2.02 (1.41–2.88)	< 0.001
Weight, kg	0.98 (0.96–0.99)	< 0.001		
Serum urea nitrogen, mg/dL	1.17 (1.13–1.21)	< 0.001		
Hypertension	1.78 (1.35–2.36)	< 0.001	1.45 (1.01–2.08)	0.042
Anemia	2.02 (1.56–2.63)	< 0.001		
Coronary artery disease	2.33 (1.29–4.20)	0.005		
Mild CKD vs non-CKD	2.87 (2.04–4.05)	< 0.001	2.23 (1.46–3.40)	< 0.001
Moderate CKD vs non-CKD	4.92 (3.27–7.40)	< 0.001	3.39 (2.05–5.62)	< 0.001
Severe CKD vs non-CKD	14.46 (8.70–24.05)	< 0.001	6.95 (3.48–13.90)	< 0.001
LVEF < 40	2.36 (1.65–3.36)	< 0.001		
Acute myocardial infarction	3.50 (2.66–4.60)	< 0.001	3.24 (2.29–4.58)	< 0.001
PCI	1.56 (1.10–2.20)	0.012		

Abbreviations: *OR* odds ratio, *HF* heart failure, *CKD* chronic kidney disease, *LVEF* left ventricular ejection fraction, *PCI* Percutaneous coronary intervention

### PAR of risk factors of CI-AKI

**Fig. 2:**
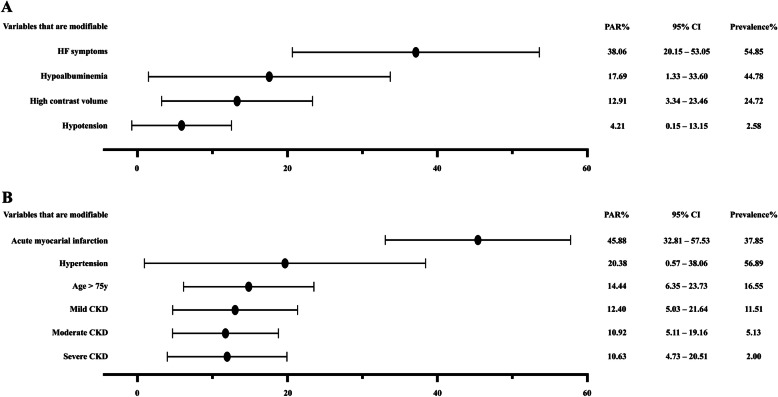
**a**: Population attributable risks of the risk factors of interest; **b**: Population attributable risks of the unmodifiable risk factors. Abbreviations: PAR: population attributable risk; HF: heart failure; CKD: chronic kidney disease

Among the four risk factors of interest of CI-AKI, the prevalence was lowest for hypotension (2.58%) and highest for HF symptoms (54.85%). The PAR was highest for HF symptoms (38.06, 95% CI: 20.15–53.05%), followed by hypoalbuminemia (17.69, 95% CI: 1.33–33.60%) and high contrast volume (12.91, 95% CI: 3.34–23.46%), and it was the lowest for hypotension (4.21, 95% CI: 0.15–13.15%) (Fig. 2a).

As for the other risk factors that were not modifiable, the PAR of AMI was 45.88% (95% CI: 32.81–57.53%), and it was 20.38% (95% CI: 0.57–38.06%) for hypertension, 14.44% (95% CI: 6.35–23.73%) for age > 75, 12.40% (95% CI: 5.03–21.64%) for mild CKD, 10.92% (95% CI: 5.11–19.16%) for moderate CKD, 10.63% (95% CI: 4.73–20.51%) for severe CKD (Fig. 2b).

## Discussion

Our study was the first one to estimate the proportion of CI-AKI attributed to four risk factors (HF symptoms, hypoalbuminemia, hypotension, and high contrast volume) that are commonly documented in cardiovascular patients and are potentially modifiable with population-level changes in operation strategy and pharmacological therapy. The highest PAR was found for HF symptoms, followed by hypoalbuminemia, high contrast volume and hypotension.

In our cohort, the incidence of CI-AKI was 7.19%, which was similar to the previous result regarding patients undergoing selected or emergent procedures [9]. In our analyses, we also found that patients with CI-AKI had a higher 10-year mortality than those without CI-AKI, which was a further exploration of previous results [3]. This finding highlights the necessity for new strategies to control several potentially modifiable risk factors, as eliminating these risk factors may cause a great reduction in the incidence of CI-AKI.

Our results indicated that HF symptoms was associated with 38.06% of the CI-AKI cases, which was the highest among the four modifiable risk factors. Based on this finding, physicians may like to find out whether heart function improving interventions before the procedure, such as dopamine and recombinant human brain natriuretic peptide (rhBNP), may help reducing the incidence of CI-AKI. In a placebo-controlled, randomized trial, Zhang et al. [14] assigned 149 acute myocardial infarction patients with HF symptoms undergoing emergency PCI to receive rhBNP or placebo. They found that periprocedural use of rhBNP could further promote the recovery of renal function and decrease the occurrence of CI-AKI. Further large, high-quality studies regarding heart function improving interventions to prevent worsen renal function are warranted.

Our study indicated that hypoalbuminemia had the second highest PAR for CI-AKI, which was somewhat unexpected. One possible explanation for our findings is that the patients in our cohort have a relatively high prevalence of hypoalbuminemia (44.78%), whereas it has also been reported by some previous studies, especially in patients with HF symptoms [15, 16]. In our study, more than half of the patients had HF symptoms. In addition, a previous study reported that the decline in albumin level appears to be caused by malnutrition and health-related factors with lower household incomes, which may be another reason for the high proportion of hypoalbuminemia in patients with cardiovascular disease in developing countries [17, 18]. The association between hypoalbuminemia and CI-AKI has been reported by some studies [19, 20]. Pooled analysis demonstrated that patients with hypoalbuminemia exhibited a higher CI-AKI rate (OR = 3.09, 95% CI = 1.44–6.64, *P* = 0.004) [21]. Our finding of hypoalbuminemia as an independent risk factor for CI-AKI was consistent with these studies. Although the mechanism of hypoalbuminemia in the occurrence and development of CI-AKI has not been fully elucidated, possible underlying mechanisms linking CI-AKI and hypoalbuminemia may be endothelial dysfunction, oxidative stress and inflammation predisposing to CI-AKI [22–24]. In addition, reducing the incidence of acute kidney injury (AKI) by improving hypoalbuminemia has been proven effective by some studies. In a randomized, double-blind trial, Lee et al. [25] found that patients with hypoalbuminemia administered 20% human albumin before the operation demonstrated a lower incidence of postoperative AKI than those administered an equal volume of saline (13.7% vs. 25.7%; RR (95% CI) = 0.533 (0.296–0.961); *P* = 0. 048). Our findings suggest that a substantial proportion (17.69%) of CI-AKI can be attributed to hypoalbuminemia alone, indicating that interventions that improve hypoalbuminemia have the potential to eliminate a large proportion of CI-AKI in patients undergoing CAG.

Our research also showed that high contrast volume and hypotension were independent risk factors of CI-AKI, which was consistent with previous studies [1, 11] and can explain 12.91 and 4.21% of CI-AKI cases, respectively. The third-place ranking of high contrast volume among the four risk factors of interest was not unexpected. Recent evidence from cohort studies and meta-analyses demonstrates that we may overestimate the CI-AKI risk conferred by exposure to contrast agents. A meta-analysis involving 25,950 patients who had intravenous administration of iodinated contrast material showed that the risk of CI-AKI was not significantly associated with contrast exposure (RR 0.79, 95% CI 0.62–1.02; *P* > 0.05) [26]. Even in critically ill patients, iodinated contrast medium exposure does not significantly increase the incidence of AKI [27]. The OR of hypotension was the highest among the four modifiable risk factors, while its prevalence was the lowest (2.58%), which made its PAR rank last among all risk factors of interest. The prevalence of hypotension in our cohort was lower than previous results [11], which may be due to the differences between patients.

Our study had some limitations. First, it was an ancillary study of an observational cohort conducted in a single center located in south China, so the prevalence of the risk factors may not be representative enough. However, this is one of the largest prospective CI-AKI cohorts, and PAR can only be calculated based on observational data. Moreover, our sample included patients at various levels of risk of CI-AKI, which made the results more generalizable. Second, risk factors of interest in this study could not cover all known and unknown CI-AKI risk factors, though the screening process was based on statistical methods, clinical importance and the results of previous high-quality studies. Third, since this was an observational study, we can only suggest that modifying these risk factors may lead to a lower incidence of CI-AKI rather than prove it. The value our results add is in providing these cost-effective targets for further intervention trials. Finally, our definition of CI-AKI may be less popular than others, though its association with long-term mortality had been proven by multivariable Cox regression.

## Conclusions

The four risk factors of interest (e.g., HF symptoms, hypoalbuminemia) could be important and cost-effective targets for prevention and treatment strategies to reduce the risk of CI-AKI. Future exploration of CI-AKI prevention strategies targeting these modifiable risk factors is warranted. In addition, our results must be interpreted with caution, as the prevalence of these risk factors may be different between regions. Larger numbers would be needed to draw more definite conclusions and apply the findings to clinical practice.

## Supplementary information

**Additional file 1.** Selection of the definition of contrast-induced acute kidney injury.

**Additional file 2.** Landmark analysis discriminating between all-cause mortality before and after 90-days of follow-up.

## Data Availability

The datasets generated and analysed during the current study are not publicly available due the institution policy but are available from the corresponding author on reasonable request.

## Abbreviations

CI-AKI: Contrast-induced acute kidney injury
MARCE: Major adverse renal and cardiovascular events
ESC: European society of cardiology
EACTS: European association for cardio-thoracic surgery
RRTs: Renal replacement therapies
PAR: Population attributable risk
CAG: Coronary angiography
PCI: Percutaneous coronary intervention
HF: Heart failure
Scr: Serum creatinine
ALB: Serum albumin
ORs: Odds ratios
CAD: Coronary artery disease
AMI: Acute myocardial infarction
AKI: Acute kidney injury
rhBNP: Recombinant human brain natriuretic peptide
