# Supplementary material for: Population attributable risk estimates of risk factors for contrast-induced acute kidney injury following coronary angiography: a cohort study
Source: BMC Cardiovasc Disord. 2020 Jun 12;20:289. doi: 10.1186/s12872-020-01570-6 (PMC7291532; doi:10.1186/s12872-020-01570-6)
Supplement: Supplementary file 1 — Additional file 1. Selection of the definition of contrast-induced acute kidney injury. [file 12872_2020_1570_MOESM1_ESM.pdf]

Selection of the definition of contrast-induced acute kidney injury

Method

The selection of the definition of contrast-induced acute kidney injury (CI-AKI) was based on the prognostic value which was evaluated using the population-attributable risk (PAR). PAR represents the proportion of cases in a population that would not have occurred in the absence of a risk factor.[1]

To identify the most prognostically valuable definition of CI-AKI, a comparison between the following 4 different definitions through PARs was performed: (1) CI-AKI_A: defined as serum creatinine elevation ≥ 0.3 mg/dL or 50% from baseline within the first 72 hours following the procedure; (2) CI-AKI_B: ≥ 0.5 mg/dL from baseline; (3) CI-AKI_C: ≥25% from baseline; (4) CI-AKI_D: ≥0.5 mg/dL or 25% from baseline.

PARs were calculated using the equation: PAR=P (HR-1)/[1+P (HR-1)], where P is the prevalence of CI-AKI under different definitions in our database. [2]

The association between CI-AKI and long-term all-cause mortality were explored by fitting a multivariable COX regression model adjusting for potential confounders (e.g. age, heart rate, heart function, renal function, medication and so on). The adjusted risk factors were selected based on univariable COX regression analysis, previous studies and clinical importance.[3, 4] Four multivariate Cox proportional hazard regression models were performed respectively for four different definitions of CI-AKI. A two-sided probability value < 0.05 was considered significant. All data analyses were conducted with R software (version 3.6.2; R Foundation for Statistical Computing,

Vienna, Austria).

Result

Among the four definitions of CI-AKI, the prevalence was highest for CI-AKI_D (12.20%), followed by CI-AKI_C (12.03%), CI-AKI_A (7.19%) and CI-AKI_B (3.28%).

During the median follow-up period of 7.41 (6.21;8.27) years, 586 all-cause death occurred. In patients with CI-AKI_A, 79 (31.85%) patients died during the follow-up period. In patients with CI-AKI_B, the number was 49 (43.36%). For patients with CI-AKI_C and CI-AKI_D, 98 (23.61%) and 102 (24.23%) death occurred respectively.

In univariable COX regression analysis, all the four definitions were associated with long-term death: CI-AKI_A (HR: 2.61, 95%CI: 2.06-3.31, P<0.001) , CI-AKI_B (HR: 4.15, 95%CI: 3.10-5.56, P<0.001) , CI-AKI_C (HR: 1.68, 95%CI: 1.35-2.09, P<0.001) , CI-AKI_D (HR: 1.75, 95%CI: 1.41-2.17, P<0.001). (Table 1)

In multivariable COX regression analysis, after adjusting for age, gender, heart rate, chronic kidney disease, heart failure, hypertension, peri-procedure hypotension, acute myocardial infarction, anemia, diabetes, high sensitive C reactive protein, angiotensin-converting enzyme inhibitor/angiotensin-receptor blockers and β blocker, all the 4 definitions were significantly associated with long-term death: CI-AKI_A (HR: 1.72, 95%CI: 1.27-2.33, P<0.001), CI-AKI_B (HR:2.24, 95%CI:1.52-3.28, P<0.001), CI-AKI_C (HR:1.32, 95%CI:1.01-1.72, P=0.038), CI-AKI_D (HR:1.36, 95%CI:1.05-1.76, P=0.021).

In addition, among the 4 definitions, CI-AKI_A (≥0.3 mg/dL or 50% from baseline)

had the highest PAR (4.92%), followed by CI-AKI$_D$ (4.21%), CI-AKI$_B$ (3.90%) and CI-AKI$_C$ (3.71%).

After all, we have chosen CI-AKI$_A$ (≥0.3 mg/dL or 50% from baseline) as the definition of the current study.

Table 1 Univariable Cox regression analysis for risk factors of long-term mortality

	HR	95%Cl		P value
Age	1.01	1.00	1.02	0.027
Gender (Female vs Male)	0.95	0.78	1.15	0.609
Weight, kg	1.00	0.99	1.00	0.401
Heart rate, bmp	1.01	1.01	1.02	<0.001
SBP	1.00	1.00	1.00	0.632
Chronic heart failure*	1.31	1.11	1.55	0.002
Chronic kidney disease	1.87	1.56	2.24	<0.001
Hypertension	1.04	0.88	1.23	0.618
Peri-hypotension	2.56	1.73	3.80	<0.001
AMI	1.33	1.13	1.57	<0.001
Anemia	1.22	1.03	1.45	0.024
Diabetes	1.18	0.98	1.42	0.075
HS-CRP, mg/L	1.01	1.00	1.01	<0.001
ACEI/ARB	0.68	0.55	0.85	<0.001
β blocker	0.65	0.53	0.80	<0.001
Diuretics	1.68	1.39	2.02	<0.001
IABP	3.03	2.25	4.09	<0.001
CI-AKI$_A$	2.61	2.06	3.31	<0.001
CI-AKI$_B$	4.15	3.10	5.56	<0.001
CI-AKI$_C$	1.68	1.35	2.09	<0.001
CI-AKI$_D$	1.75	1.41	2.17	<0.001

Abbreviations: SBP: systolic blood pressure; AMI: acute myocardial infarction; HS-CRP: high-sensitivity C-reactive protein; ACEI: angiotensin-converting enzyme inhibitor; ARB: angiotensin-receptor blockers; IABP: intra-aortic balloon pump; CI-AKI: contrast-induced acute kidney injury;

*Chronic heart failure: defined as New York Heart Association (NYHA) class > I/Killip

class > I on presentation or left ventricular ejection fraction <40%.

Reference

1. Spiegelman D, Hertzmark E, Wand HC: **Point and interval estimates of partial population attributable risks in cohort studies: examples and software**. *Cancer Causes Control* 2007, **18**(5):571-579.
2. Willey JZ, Moon YP, Kahn E, Rodriguez CJ, Rundek T, Cheung K, Sacco RL, Elkind MS: **Population attributable risks of hypertension and diabetes for cardiovascular disease and stroke in the northern Manhattan study**. *J Am Heart Assoc* 2014, **3**(5):e001106.
3. Roe MT, Chen AY, Thomas L, Wang TY, Alexander KP, Hammill BG, Gibler WB, Ohman EM, Peterson ED: **Predicting long-term mortality in older patients after non-ST-segment elevation myocardial infarction: the CRUSADE long-term mortality model and risk score**. *American heart journal* 2011, **162**(5):875-883.e871.
4. Shuvy M, Beeri G, Klein E, Cohen T, Shlomo N, Minha S, Pereg D: **Accuracy of the Global Registry of Acute Coronary Events (GRACE) Risk Score in Contemporary Treatment of Patients With Acute Coronary Syndrome**. *Can J Cardiol* 2018, **34**(12):1613-1617.
