# Supplementary material for: Population attributable risk estimates of risk factors for contrast-induced acute kidney injury following coronary angiography: a cohort study
Source: BMC Cardiovasc Disord. 2020 Jun 12;20:289. doi: 10.1186/s12872-020-01570-6 (PMC7291532; doi:10.1186/s12872-020-01570-6)
Supplement: Supplementary file 2 — Additional file 2. Landmark analysis discriminating between all-cause mortality before and after 90-days of follow-up. [file 12872_2020_1570_MOESM2_ESM.pdf]

Figure S1. Landmark analysis discriminating between all-cause mortality before and after 90-days of follow-up

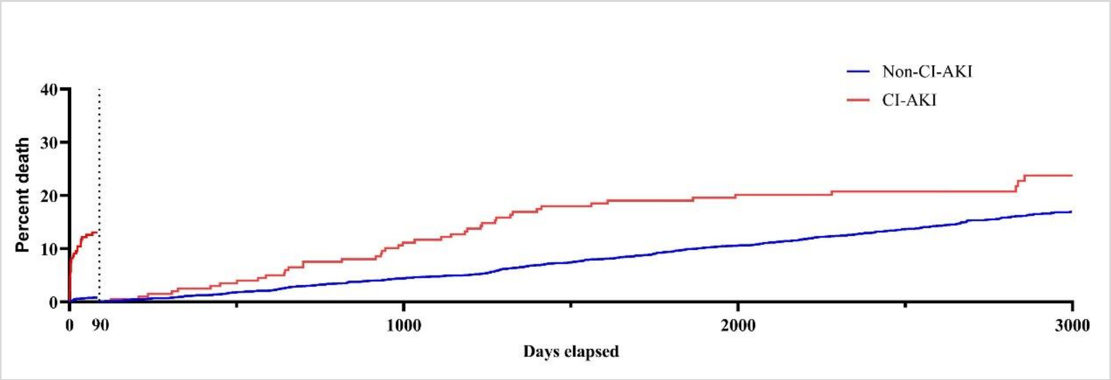

Before 90-days: log-rank P < 0.01; After 90-days: log-rank P < 0.01
